# A new 1D Mn(II) coordination polymer: Synthesis, crystal structure, hirshfeld surface analysis and molecular docking studies

**DOI:** 10.1016/j.heliyon.2024.e29565

**Published:** 2024-04-20

**Authors:** Atash V. Gurbanov, Fateme Firoozbakht, Nafiseh Pourshirband, Paria Sharafi-Badr, Payam Hayati, Bagher Souri, Fazlolah Eshghi, Werner Kaminsky, Ghodrat Mahmoudi, Francis Verpoort, Zohreh Mehrabadi

**Affiliations:** a Centro de Química Estrutural, Institute of Molecular Sciences, Instituto Superior Técnico, Universidade de Lisboa, Av. Rovisco Pais, 1049-001 Lisboa, Portugal; b Excellence Center, Baku State University, Z. Khalilov Str. 23, AZ 1148 Baku, Azerbaijan; c Department of Chemistry, University of Isfahan, Isfahan 81746-73441, Iran; d Department of Chemistry, Shahreza Branch, Islamic Azad University, P.O. Box 311-86145, Shahreza, Isfahan, Iran; e Department of Pharmacognosy and Pharmaceutical Biotechnology, School of Pharmacy, Iran University of Medical Sciences, Tehran, Iran; f Organic and Nano Group (ONG), Department of Chemistry, Iran University of Science and Technology (IUST), PO Box 16846-13114, Tehran, Iran; g Department of Chemistry, Faculty of Sciences, University of Sistan and Baluchestan, Zahedan, Iran; h Department of Chemistry, College of Sciences, Shiraz University, Shiraz, Iran; i X-ray Crystallography Laboratory, University of Washington, United States; j Department of Chemistry, Faculty of Science, University of Maragheh, P.O. Box 55136-83111, Maragheh, Iran; k Chemistry Department, Faculty of Engineering and Natural Sciences, Istinye University, Sarıyer, Istanbul 34396, Turkey; l State Key Laboratory of Advanced Technology for Materials Synthesis and Processing, Wuhan University of Technology, Wuhan 430070, China; m Department of Chemistry, Firoozabad Branch, Islamic Azad University, Firoozabad, Iran; n Western Caspian University, Istiqlaliyyat Street 31, AZ 1001, Baku, Azerbaijan

**Keywords:** Supramolecular chemistry. metal-organic coordination polymers (MOCP). solvothermal. sonochemical ultrasonic-assisted. density functional theory (DFT)

## Abstract

The synthesis of novel metal-organic coordination polymers (MOCP) with the chemical formula [Mn_2_L (SCN)_2_(OH)_2_]_3_·CH_3_OH [L = 1,5-bis(pyridine-4-ylmethylene) carbonohydrazide] {**1**} was accomplished using two different techniques: solvothermal and sonochemical ultrasonic-assisted. An investigation was carried out to examine the impact of various factors such as reaction time, sonication power, temperature, and reactant concentration on the morphology and size of the crystals. Interestingly, it was found that sonication power and temperature did not affect the crystals’ morphology and size. To further analyze the prepared microcrystals of MOCPs, SEM was utilized to examine their surface morphology, and XRD, elemental evaluation composition. The identification of the functional groups present in the prepared Mn-MOCPs was accomplished through the utilization of FT-IR spectroscopy. Subsequently, the calcination of **1** in an air atmosphere at 650 °C led to the formation of Mn_3_O_4_ nanoparticles. The geometric and electronic structure of the MOCPs was evaluated using density functional theory (DFT). The utilization of molecular docking methodologies demonstrated that the best cavity of the human androgen receptor possessed an interaction energy of −116.3 kJ mol^−1^. This energy encompassed a combination of both bonding and non-bonding interactions. The Results showed that steric interaction and electrostatic potential are the main interactions in AR polymer and Mn(II). These interactions in the defined cavity indicated that this polymer could be an effective anti-prostate candidate, because AR is involved in the growth of prostate cancer cells, and these interactions indicated the inhibition of prostate cancer cell growth.

## 1 Introduction

Supramolecular chemistry encompasses a diverse array of molecular assembly states, which are governed by an assortment of non-covalent interactions [[1], [2], [3], [4], [5]]. These interactions include coordination bonds [6,7], ionic interactions [8,9], hydrogen bonds [10,11], π-π stacking [12,13], and host-guest interactions [[14], [15], [16]]. The interplay of these interactions plays a vital role in orchestrating the formation of intricate architectures and dictating their properties at both the microscopic and macroscopic levels. Owing to its potential and applicability, supramolecular chemistry has been extensively explored, leading to the development of several subareas [[17], [18], [19], [20], [21]]. These complexes have applications in bioinorganic chemistry, separations, catalysis, hydrometallurgy, and material science. Metal coordination complexes with popular pseudohalides like OCN^−^, SCN^−^, SeCN^−^, and N_3_^−^ are studied for their structural aspects and unique properties. Thiocyanate-related complexes exhibit diverse binding chemistry and form versatile structural bridging motifs with metal ions. Coordination polymers, especially those with SCN^−^ -ligands, are notable in coordination chemistry for their intriguing architectural frameworks and diverse bonding functionalities seen in X-ray crystal structures [[22], [23], [24], [25]]. Analyzing coordination polymer analogs through crystallography helps researchers understand their structural relationships, similarities, and differences, aiding in exploring their properties and applications. Different analog types include isomorphous, topological, structural, and functional analogs, each with unique crystallographic features and comparisons. Isomorphous analogs share the same crystal structure but have different chemical compositions. Topological analogs exhibit similar connectivity patterns with variations in ligands or metal ions. Structural analogs display similar overall structures with differences in ligand or metal ion arrangements [[26], [27], [28]].

In recent decades, there has been a substantial focus on the synthesis and characterization of MOCPs, metal-organic frameworks (MOFs), and coordination polymers (CPs) [[29], [30], [31], [32], [33], [34], [35], [36], [37], [38], [39]]. The acquisition of precise single-crystal X-ray diffraction measurements for MOCPs typically requires the utilization of diverse processes in wet solution chemistry and/or solvothermal techniques. In numerous instances, longer reaction times are preferred for diffusion methods [40]. The potential industrial applications of MOCPs are realized by employing solvothermal conditions, which involve the reaction of framework building blocks at high temperatures and pressures. This process allows for the synthesis of MOCPs with desirable properties for various industrial applications. New synthetic techniques have been developed to produce these compounds on a larger scale [41]. In recent years, newly developed techniques—such as chemical mechanics [[42], [43], [44], [45]], microwave (MW) [[46], [47], [48], [49]], and electrochemical synthesis [50,51] have aimed to increase product yield by significantly reducing synthesis time. Additionally, the sonochemical method for the fabrication of MOCPs is emphasized as both a conventional and a novel synthetic approach that offers simplicity, convenience, and controllability when compared to other strategies [[52], [53], [54], [55], [56], [57], [58]]. Finally, the use of ultrasound (US) for the synthesis of MOCPs is highlighted as one of the most powerful tools in the preparation of CPs and MOFs [[59], [60], [61], [62], [63], [64], [65], [66], [67]]. The research primarily focuses on the preparation of single crystals of manganese (II) coordination polymer (MOCP) and nanostructures of Mn-MOCPs ([Mn_2_ L (SCN)_2_(OH)_2_]_3_·CH_3_OH) using a straightforward sonochemical method. This method offers a simple and efficient approach to synthesizing both the single crystals and nanostructures of Mn-MOCPs. Furthermore, the research assesses the influence of fundamental parameters, such as reaction time, ultrasonic power, temperature, and reactant concentration on the structural characteristics of the synthesized Mn-MOCPs. Synthesized polymers such as Metallodrugs may serve as potential anti-cancer agents. Therefore, we conducted an in silico study to explore the anti-prostate cancer properties of this polymer.

## 2 Materials and methods

The materials used in this study were sourced from Sigma-Aldrich and were used without the need for further purification. The research involved the utilization of several analytical and characterization techniques: Elemental analyses were performed using a Heraeus Analytical Jena Multi EA 3100 CHNO analyzer. FT-IR spectra were recorded using a JASCO 680-PLUS spectrophotometer. SEM images were obtained using a FEI Quanta 650 FEG instrument. XRD measurements were conducted with a Philips X'pert diffractometer equipped with a monochromated CuKα radiation source. Ultrasonic irradiation was employed with the aid of three devices: an Elmasonic (Elma) S40H homogenizer, an ultrasonic homogenizer-UP 400-A (IRAN), and an electrothermal 9100 apparatus for determining melting points. For single-crystal X-ray diffraction analysis of compound **1**, a Bruker-Nonius KappaCCD diffractometer equipped with an Oxford Cry-stream 700 apparatus was utilized. The analysis utilized graphite monochromated MoKα radiation (λ = 0.71073 Å) at a temperature of 173 (2) K. The SADABS program was employed for data reduction and correction for semi-empirical absorption [68]. The SIR97 program was used to solve the structure [69]. Refinement was carried out using the full-matrix least-squares method on F2 with the support of the SHELXL-2016/6 program [70]. The WinGX program [71] provided additional assistance. The PLATON program was utilized for molecular graphics [72]. Hirshfeld surface analysis was carried out using CrystalExplorer17 software [73]. Furthermore, simulated XRD patterns based on single-crystal data were obtained using the Mercury software [74].

In the theoretical part, we conducted an in silico study involving quantum mechanics (QM) and molecular mechanics (MM) sections. In the QM part, we used the density functional theory (DFT) method. for convenience, a level of theory (method/basis set) was selected for structure optimization. The DFT calibration method was employed for this purpose. This approach entailed comparing geometric parameters of optimized structures at various levels of theories to experimental data such as bond lengths. In this study, the calculated Mn(II) polymer bond lengths were compared to x-ray crystallography experimental data in Table 1. A level of theory with minimal deviation from experimental data was considered a reliable method/basis for theoretical studies, known as the DFT calibration method [75].

**Table 1:** Calculated and experimental bond lengths of the Mn(II) polymer in Ǻ for theoretical study in all method/basis sets.

Method/basis set	C_(1)_–N_(1)_	C_(1)_–H_(1)_	Mn_(1)_–O_(2)_
PW91/Lanl2dz	1.311	0.906	2.191
TPSS/Lanl2dz	1.309	0.912	2.189
B97/Lanl2dz	1.320	0,917	2.198
M06/Lanl2dz	1.305	0.909	2.184
Experimental	1.330	0.930	2.208

According to this table, the B97/LANL2DZ level of theory has a low deviation from x-ray crystallography data. This method is a combination of hybrid DFT (B97) and Los Alamos National Laboratory double-ζ (which is a widely used effective core potential (ECP)-type basis set), was chosen as the appropriate method to Refs. [76,77]. So, Geometric and electronic investigation was carried out using this method in the Gaussian 09 package [78].

In the MM part, we conduct molecular docking studies using Molegro Virtual Docker (MVD) 6.01 to assess the affinity of Mn(II) polymer toward the human androgen receptor [79]. The docking procedure utilized the MolDock algorithm and Piecewise Linear Potential (PLP) expansion [80]. The following parameters were used: MolDock scoring function iterations: 1500, simplex evolution size: 50, and at least 10 runs. The PDB file with ID 2oz7 [81] for the human androgen receptor was acquired from the Research Collaborative for Structural Bioinformatics (RCSB) Protein Data Bank (http://www.rcsb.org). During geometry minimization, the best cavity was identified based on specific Cartesian coordinates, with a radius of 15 Å, volume of 1438.2 Å³, and surface area of 2420.5 Å^2^. In the molecular docking process, the flexibility of the side chain of the amino acid residues within the cavity was taken into consideration. The applied parameters included a tolerance of 1.00 and a power of 0.90, with the maximum global minimization step set to 1500. Furthermore, the Simplex and MolDock evolution grid score parameters were configured with 300 steps, a neighbor distance factor of 1.00, and a grid resolution of 0.30 Å.

### 2.1 Synthesis of ligand 1,5-bis (pyridine-4-yl methylene) carbon-hydrazide and compound 1 as a single crystal structure

Synthesis of 1,5-bis (pyridine-4-yl methyl ene) carbon-hydrazide: The Schiff base ligand was prepared by the method described below and used without further purification. All other reagents and solvents used for synthesis and analysis were commercially available and used as received. An ethanol (60 ml) mixture of 4-pyridine carboxaldehyde (1.07 g 10 mmol) and carbohydrazide (0.45 g.5 mmol) with 15 drops of CH_3_COOH was refluxed in an oil bath for 6 h. After the volume of the reaction mixture was reduced to about 15 ml white precipitate started to form. Insoluble product was removed by filtration, washed with ice-cold ethanol (3 × 2 ml), and dried on air to give pure product (1.045 g) with a yield of 78 %. Anal. Calc. for C_13_H_12_N_6_O (268.11) (%): C 58.20, H 4.51 and N 31.33. found: C 58.34, H 4.59, and N 31.19. IR data (KBr, cm^−1^) *ν*: 3384 (NH), 3071 (Ar-CH), 22,930 (Alip.-CH), 1688 (C]O), 1527 (CH]N), 1457 (CH]N)_pyridine_.

The synthesis of **1**, 1,5-bis (pyridine-4-ylmethylene) carbon-hydrazide, was carried out using the solvothermal method. 256 mg (1 mmol) of 1,5-bis-(pyridine-4-yl-methylene) carbonic hydrazide was dissolved in 15 mL of distilled methanol. Then 194 mg (2 mmol) of KSCN was added to it and the resulting mixture was vigorously stirred for 30 min at room temperature. 178 mg (1 mmol) of Mn(NO_3_)_2_ was slowly added to the reaction mixture and the resulting reaction mixture was transferred to a stainless steel container with a Parr Teflon cover. The container was placed at a temperature of 85 °C for three days. After heating, the mixture was gradually cooled to room temperature, the blue crystals of compound **1** were collected from the mixture, and the filtered crystals were air-dried at room temperature. Subsequently, the crystals were subjected to single-crystal X-ray diffraction analysis. The analytical calculations indicated C_15_H_12_MnN_8_OS_2_CH_4_O_2_(HO) with values of C = 14.15 %, H = 0.94 %, O = 3.77 %, and a found composition of C = 14.12 %, H = 0.91 %, O = 3.45 %. Additionally, the IR spectrum for **1** revealed peaks at 3400 (b), 3140 (w), 2071 (s), 1686 (s), and 1064 (s) cm^−1^.

### 2.2 Synthesis of 1 under ultrasonic irradiation

Compound 1-1 was synthesized using the sonochemical irradiation method. The procedure involved the following steps: A high-density ultrasonic probe was submerged in a solution of Mn (NO_3_)_2_ (10 mL, 0.1 M). Following that, 10 mL of 0.1 M 1,5-bis (pyridine-4-yl methylene) carbon-hydrazide [L] and 10 mL of 0.1 M KSCN [L^/^] were added carefully. The experiment was repeated multiple times, with each repetition involving the modification of a single parameter, as detailed in Table S1. After the reaction, the resulting precipitates were filtered, washed with DMF (dimethylformamide), and dried. Analytical calculations of the synthesized compound indicated a composition of C_15_H_12_MnN_8_OS_2_CH_4_O_2_(HO), with the following elemental composition: C = 14.15 %, H = 0.94 %, O = 3.77 %. The experimental analysis yielded the following results: C = 14.10 %, H = 0.89 %, O = 3.28 %, which were close to the calculated values. Additionally, IR analysis of **1**–**1** revealed specific bands at the following wavenumbers: 3991 (b), 3151 (w), 2059 (s), 1974 (s), and 1062 (s).

### 2.3 Preparation of Mn_3_O_4_ nanoparticles

The process of synthesizing Mn_3_O_4_ nanoparticles involved transferring a certain amount of material labeled as ‘**1’** into a porcelain crucible, heating it to 650 °C in a furnace under an air atmosphere. Subsequently, after approximately 4 h, the resulting powder was cleaned with a small quantity of acetone to eliminate impurities and then dried at 70 °C for **1** h, resulting in the formation of Mn_3_O_4_ nanoparticles.

## 3 Results and discussions

### 3.1 Single crystal X-ray analysis

**Fig. 1:**
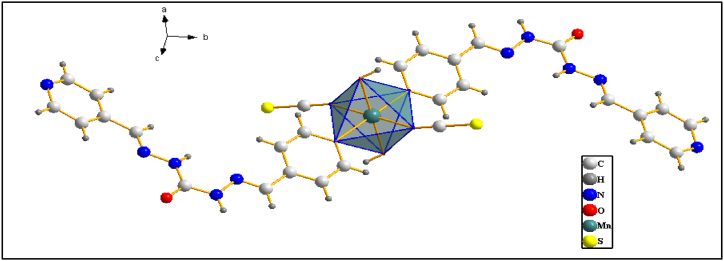
Coordination around Mn^2+^ cations in **1.**

**Fig. 2:**
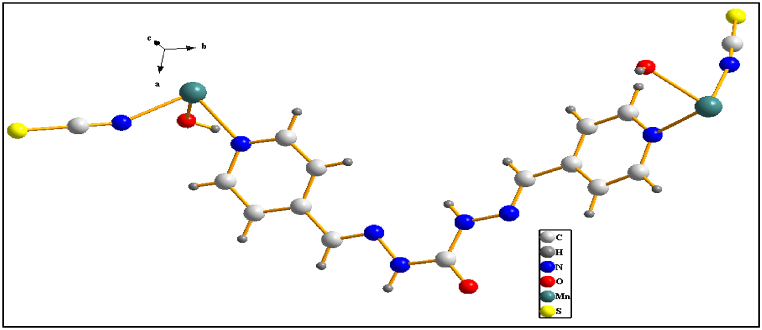
The asymmetric unit of a of **1.**

**Fig. 3:**
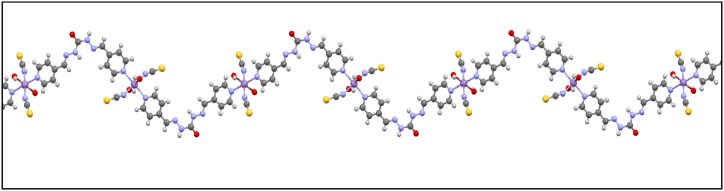
Zig-zag chain of CPC **1.**

Reaction between the organic nitrogen-donor 1,5-bis (pyridine-4-ylmethylene) carbon-hydrazide ligand (L), KSCN, and Mn(NO_3_)_2_ yielded crystalline material formulated as new 1D coordination polymer [Mn_2_L (SCN)_2_(OH)_2_]_3_·CH_3_OH (**1**). Utilizing 1,5-bis (pyridine-4-ylmethylene) carbon-hydrazide for N-donor ligand in a mixture of KSCN and Mn(NO_3_)_2_ leads to the formation of new coordination polymer **1**. The crystallographic analysis of **1** indicates that the Mn atoms exhibit coordination with the following ligands: two N atoms of two pyridine rings (Mn1–N1 distance: 2.2631 Å), two N atoms of two isothiocyanate groups (Mn1–N7 distance: 2.1846 Å), and two O atoms of two hydroxyl groups (Mn1–O2 distance: 2.2082 Å), resulting in an octahedral coordination O_4_N_2_. Detailed crystallographic information is available in Tables 2 and S1, and Fig. 1. The compound crystallized in the triclinic P_-1_ space group. The distance between the Mn^2+^ cation and the N atoms from the pyridine rings was equal, and larger than the distances between the Mn^2+^ cation and the N atoms from the isothiocyanate groups. In **1**'s asymmetric unit, there exist two Mn^2+^ cations, each binding to one 1,5-bis(pyridine-4-yl methylene) carbonohydrazide (L), one isothiocyanate group, and one O atom of the hydroxyl group. Each L ligand in **1** interacts with two Mn atoms through N atoms of pyridine rings, with distances of Mn1–N1 at 2.2136 Å and Mn2–N6 at 2.2899 Å (refer to Fig. 2). The Mn (NCS)_2_(L)_2_(OH)_2_ units are interconnected with other molecules via a bridging N atom of the pyridine ring, forming a one-dimensional coordination polymer, resulting in a zig-zag structure due to the bending structure of L (see Fig. 3).

**Table 2:** Selected bond lengths/Å for **1**.

Mn (2)–N (6)	2.289 (4)	C (3)–C (4)	1.372 (5)
Mn (2)–N (8)	2.289 (4)	C (3)–C (6)	1.458 (5)
Mn (1)—N (7)	2.185 (3)	C (4)–C (5)	1.373 (5)
Mn (1)—O (2)	2.208 (3)	C (5)–N (1)	1.322 (4)
Mn (1)—O (3)	2.186 (6)	C (6)–N (2)	1.266 (4)
C (1)–N (1)	1.330 (4)	C (7)–O (1)	1.215 (4)
C (1)–C (2)	1.375 (5)	C (7)–N (4)	1.340 (5)
C (1)–H (1)	0.93	C (7)–N (3)	1.354 (5)
C (2)–C (3)	1.367 (5)	C (8)–N (5)	1.240 (5)
C (2)–H (2)	0.93	C (8)–C (9)	1.484 (5)

**Fig. 4:**
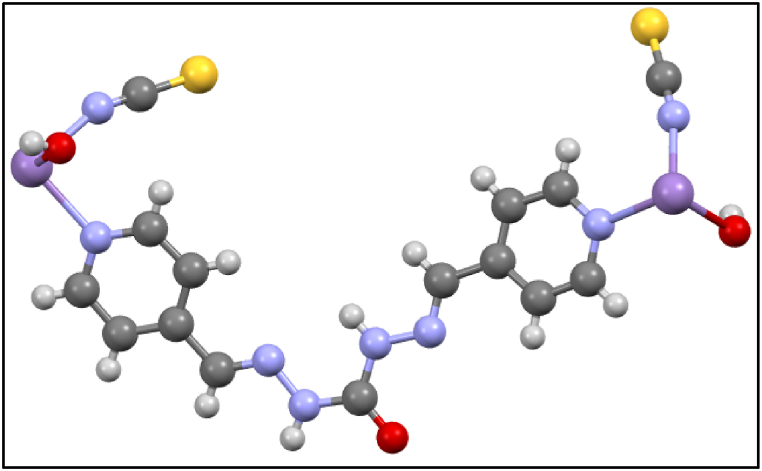
Asymmetric unit cell of **1**.

**Fig. 5:**
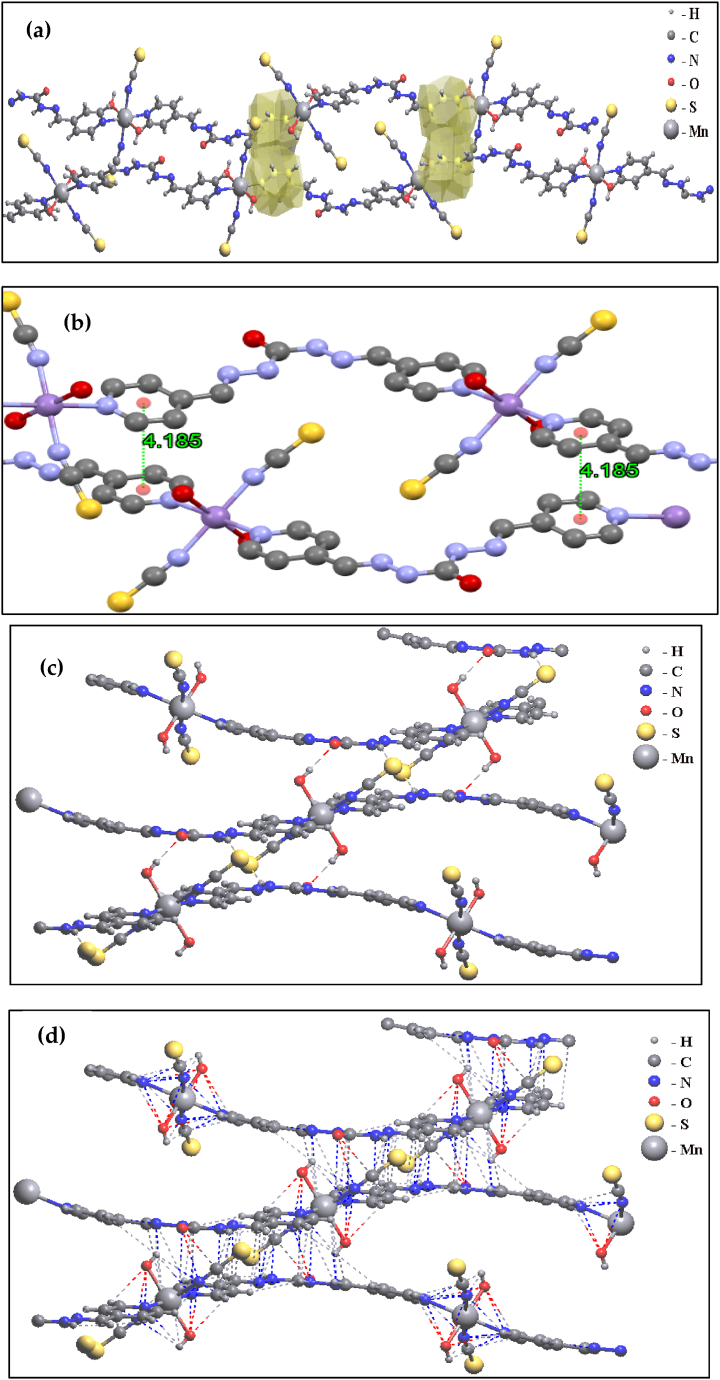
Hydrogen bound (a), van der Waals interactions (b), π – π interaction (c), and distance of two aromatic rings (d) in **1**.

The asymmetric unit cell of **1** consists of one Mn^+2^ cation, one L, two O atoms of OH, and two –NCS (Fig. 4). There is π–π interaction between aromatic rings of chains in the crystal packing of **1**, Also the distance between two aromatic rings is 4.185 Å which is pleasant to make intermolecular interaction. Each chain has hydrogen-bound contacts with other chains by O atoms (OH) and H atoms (L). On the other hand, van der Waals' interaction is revealed in Fig. 5a-d.

**Fig. 6:**
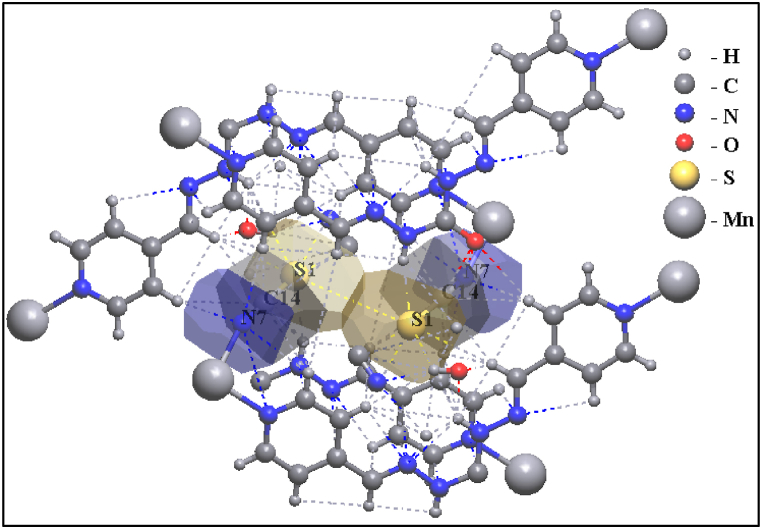
van der Waals interactions of SCN^−^ in crystal packing of **1**.

**Fig. 7:**
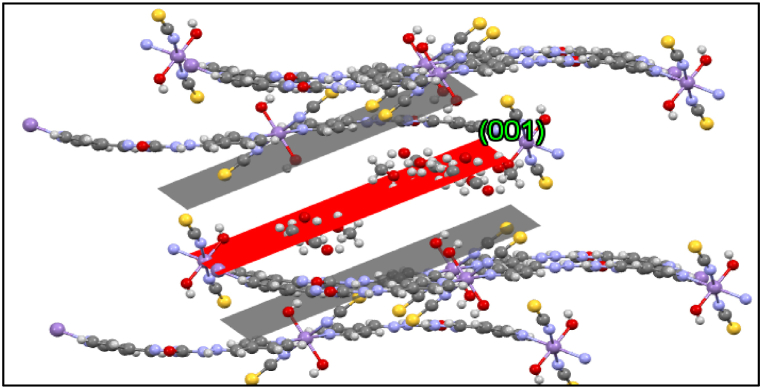
Crystal packing along the [001] for **1**.

**Fig. 8:**
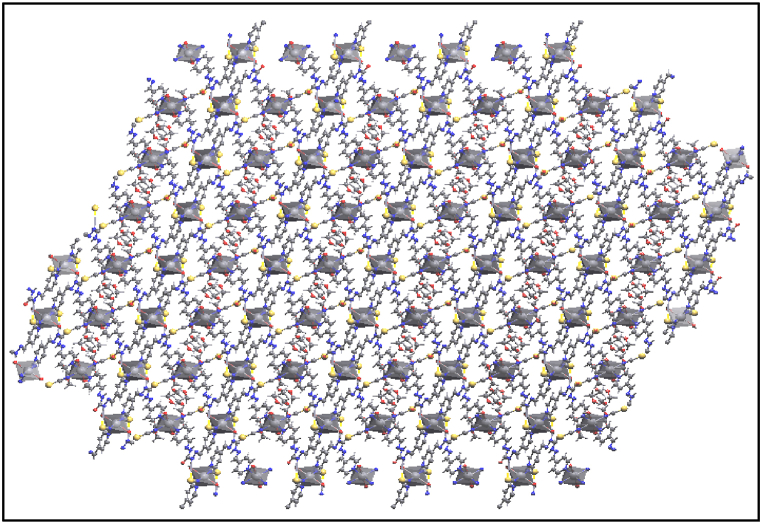
A fragment of the 3D framework in **1**.

In a solid-state network, these coordination compounds are connected by hydrogen bonding and some other intermolecular interactions leading to 3D expanded crystal packing (Fig. 5a–d). It should be noted that SCN^−^ has an important role in growing 3D framework crystal packing of **1** by intermolecular van der Waals interaction between H and C of ligand (Fig. 6.). As shown in Fig. 7, the growth of the manganese coordination compound crystal occurs along the (001) plane. The morphology of the crystal packing **1** is hexagonal and is shown in Fig. 8. In this way, the crystal structure described here as a typical compound can be considered as a shred of powerful evidence confirming the suggested structures of the titled manganese coordination compounds in current research.

### 3.2 IR spectroscopic analysis

The FT-IR spectra of **1**, as depicted in Fig. S1, exhibit characteristic stretching absorption bands for O–H, N–H, –C]O, and –C]N. The broad absorption peak at around 3400 cm^−1^ corresponds to the O–H group coordinated with the metal atom, while the absorption peak at 3140 cm^−1^ corresponds to the vibration of N–H. Additionally, the C–H in-plane bending vibration is observed in the range of 1372–1064 cm^−1^, with out-of-plane vibrations measured at 937 cm^−1^. Moreover, the vibration peaks at 680 cm^−1^ are attributed to the deformation vibration of the pyridine ring, and the sharp absorption bands at 2071 cm^−1^ are related to the SCN groups. Strong IR absorptions at 1678 cm^−1^ are observed, which were assigned to the (C]O) modes. Such a low value for the carbonyl double bonds is also a signature of the presence of an intramolecular N and O]C interaction [82].

### 3.3 PXRD analysis of 1 and Mn_3_O_4_

**Fig. 9:**
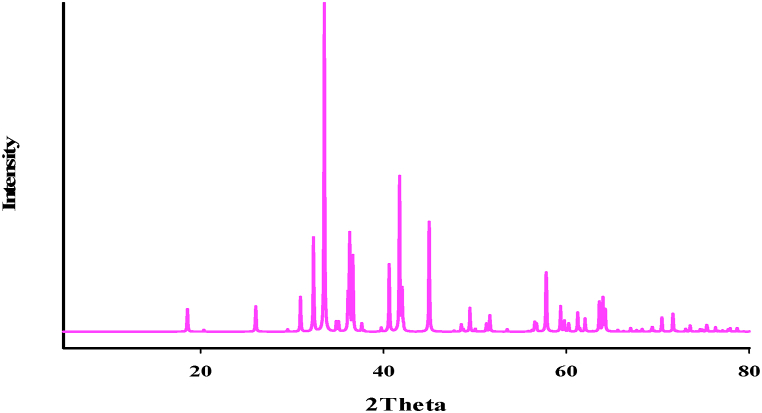
Xrd of Mn_3_O_4_.

The XRD patterns of **1,** as shown in Fig. S2, indicate good agreement with slight variations in 2θ values between the simulated XRD patterns from single-crystal X-ray data and the experimental powder X-ray diffraction patterns of **1** prepared using the sonochemical process. This suggests that the compound obtained through the sonochemical process, in the form of nano-structures, exhibits a crystal structure similar to that obtained through single-crystal diffraction. The observed differences between the XRD pattern data and the patterns simulated from single-crystal X-ray data indicate significant peak broadening. This broadening implies that the particles of **1** are of nanometer dimensions, indicating the presence of nano-sized crystalline domains in the sample. The X-ray diffraction (XRD) pattern displayed in Fig. 9 corresponds to the residue obtained when **1** was subjected to calcination at 650 °C under ambient conditions for 4 h. The observed XRD pattern closely matches the standard pattern of Mn_3_O_4_, and the lattice parameters (a = 5.7621 Å, c = 9.4696 Å, space group = *I*4_1_/*amd*, and z = 4) are by the reported data (JCPDS card number 24–0734).

Furthermore, an electron microscopy (SEM) image of the residue, obtained through the direct calcination of single crystals of **1** at 650 °C, reveals the formation of agglomerated and non-uniform spherical Mn_3_O_4_ nanoparticles. These nanoparticles exhibit a ligand-free octahedral-like morphology.

### 3.4 SEM analysis of 1 and Mn_3_O_4_

The sonochemical technique has emerged as a promising method for the production of coordination polymers. It offers several advantages, including high product yields, simple reaction conditions, and reduced waste generation compared to other methods such as layering, solvothermal, and diffusion approaches [83,84].

**Fig. 10:**
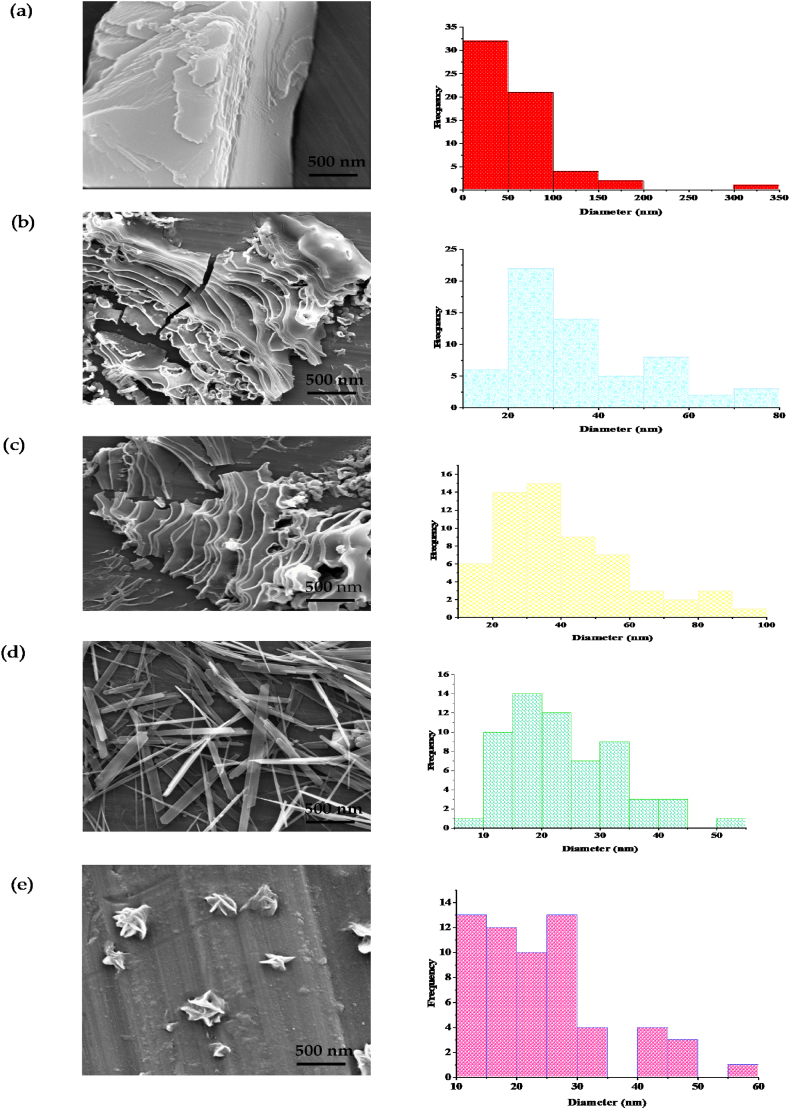
SEM images and particle size distribution histogram related to synthesized micro and nanoparticles: (a) **1** without sonochemical reaction, (b) **1** with sonochemical reaction (50 °C, reaction time 60 min, concentration of reactants 0.1 M, power 60 W), (c) same as (b) but reaction time 30 min, (d) same as (b) but with reactant concentration 0.5 M, (e) MOF-1s same as (b) but at 70 °C.

**Fig. 11:**
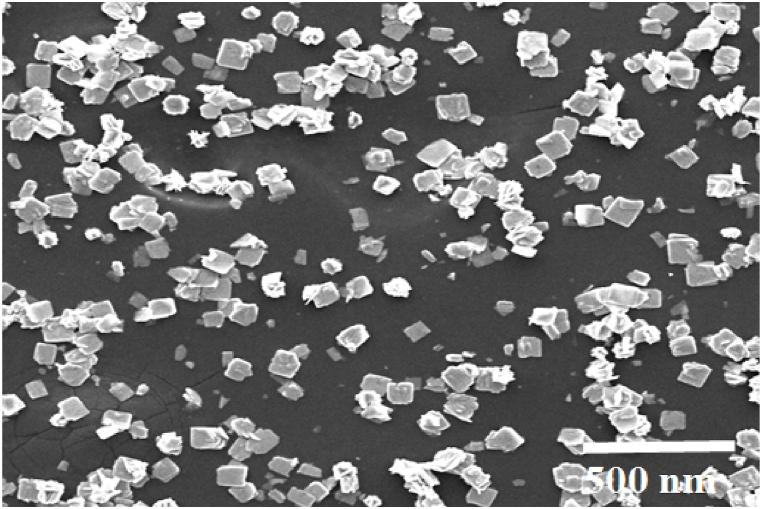
SEM images of Mn_3_O_4_ prepared from thermal decomposition of **1**.

Scanning electron microscopy (SEM) images of coordination polymer **1**, synthesized using an ultrasonic generator with a power of 60 W and initial reagent concentrations of [Mn^2^⁺] = [L] = [KSCN] = 0.1 mol L⁻^1^, reveal a mixed-shape structure consisting mainly of flake (Fig. 10a), mixed morphologies (Fig. 10b and c), rod (Fig. 10d), and flower (Fig. 10e) along with microscale agglomerates. Various reaction conditions were tested, as presented in Table 3 and Fig. 10. It was observed that the particle size increases as the reactant concentration decreases. Similarly, an increase in reaction temperature, as shown in Fig. 10b and e, also led to an increase in particle size. Under the conditions of 60 W ultrasonic irradiation, a reaction time of 60 min, a reagent concentration of 0.1 M, and a temperature of 70 °C, a less agglomerated nanostructure of 1–5 was formed (Fig. 10e). Following ultrasonic treatment, the morphology partially transformed into small rod and flower shapes (Fig. 10d and e). The SEM image of Mn_3_O_4_, on the other hand, displays agglomerated and non-uniform spherical nanoparticles (Fig. 11).

**Table 3:** Influence of reaction time, sonication power, temperature, and the concentration of reactants on the morphology and size of **1**.

Entry	Sonication power (W)	t (min)^a^	Concentration (M)^b^	T (°C)^c^	Morphology
1-1	0	60	0.1	50	Flakes (2D)
1-2	**60**	60	0.1	50	Mixed morphology
1-3	60	**30**	0.1	50	Mixed morphology
1–4	60	60	**0.5**	50	Rods morphology (1D)
1–5	60	60	0.1	**70**	Flower

a Reaction time

b Concentration of reactants.

c Temprature of reactants.

### 3.5 Hirshfeld surface analysis (HSA)

**Fig. 12:**
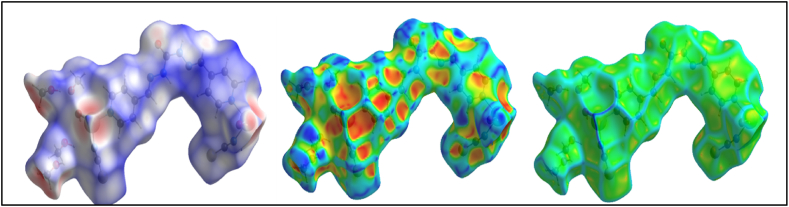
Hirshfeld surfaces of **1** were mapped using dnorm, shape index, and curvature.

**Fig. 13:**
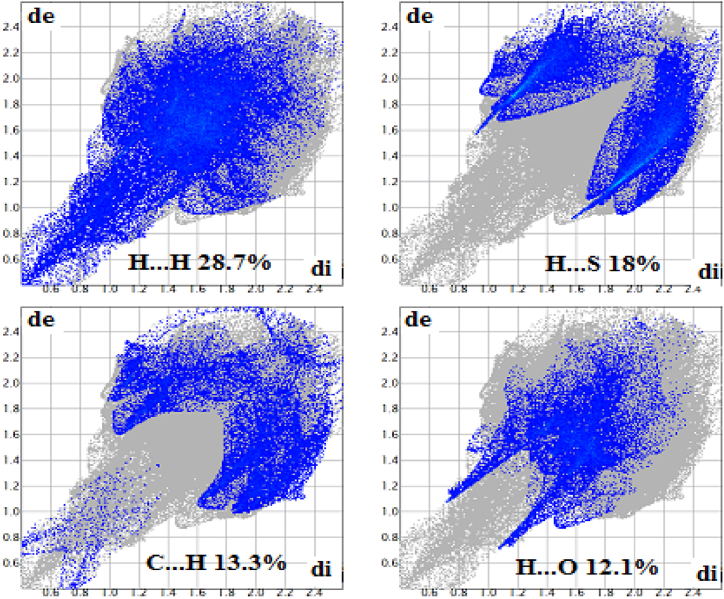
2D fingerprint plots for **1**.

To investigate various non-covalent interactions in multicomponent crystal structures for **1**, two-dimensional fingerprint diagrams and Hirshfeld surface analysis (HSA) were used. The main purpose of this analysis was to emphasize the specific characteristics of the mapped HSA and HSA dormitories. Quantitatively, various interactions and their contribution to HSA were investigated [85,86]. Hirshfeld surfaces illustrated in Fig. 12were generated using properties such as dnorm, shape index, and curvedness. These surfaces offer visual representations of the electron density distribution around the molecule. Fig. 13 summarizes the percentage contributions of different contacts (C⋯H, H⋯H, N⋯H, and S⋯H) to the HSA of the **1**. The dark red regions in the figures indicate significant O··H bonding contacts and a negative dnorm surface [87,88], suggesting the presence of strong hydrogen bonding interactions. Moreover, the intermolecular contacts were found to extend beyond the expected van der Waals radius. This suggests the presence of attractive non-covalent interactions between the molecules within the crystal structure. These interactions play a crucial role in shaping the overall structure and properties of the **1**. The H⋯H interactions were identified as weak and exhibited short spikes in the center of the fingerprint plot, indicating their significance as the most prominent contacts on the total Hirshfeld surfaces. These interactions accounted for approximately 28.7 % of the total Hirshfeld surface area for **1**.

**Fig. 14:**
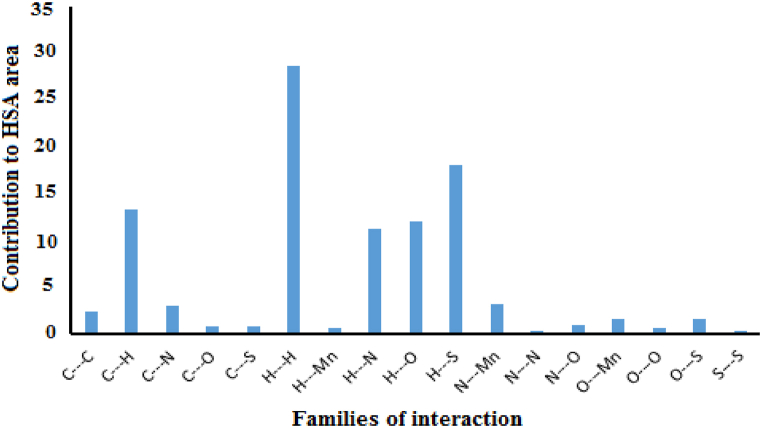
The relative contributions to the Hirshfeld surface area for **1**.

The interactions involving sulfur and hydrogen atoms, known as S⋯H/H⋯S interactions, accounted for 18 % of the total Hirshfeld surface area. These interactions contribute to the overall stability of the crystal structure. The extents of C⋯H/H⋯C interactions covered 13.3 % of **1**. These interactions involve carbon and hydrogen atoms and play a role in the crystal structure. The extents of O⋯H/H⋯O interactions covered 12.1 % of **1**. These interactions involving oxygen and hydrogen atoms, known as hydrogen bonding contacts, also contribute to the stability of the crystal structure [89,90]. The prevalence of these hydrogen bonding contacts provides compelling evidence for the stable presence of crystal structures in **1**. Fig. 14 illustrates the relative contributions of these different interactions to the Hirshfeld surfaces for the title complex. This visualization helps to understand the distribution and significance of these interactions within the crystal structure of **1**.

### 3.6 Theoretical studies

**Fig. 15:**
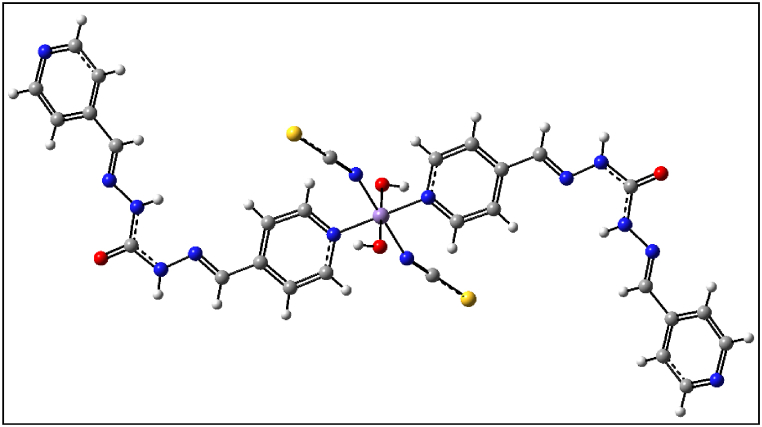
Optimized geometry structures of **1**.

**Fig. 16:**
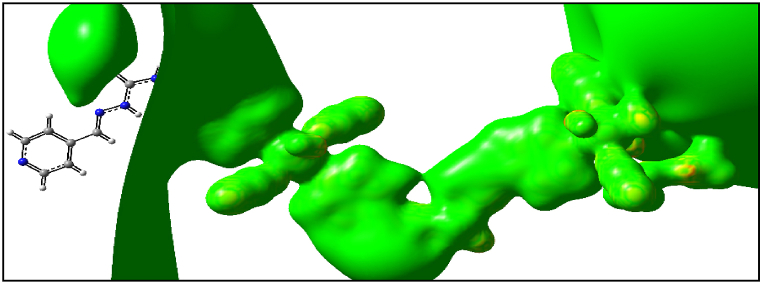
Calculated molecular electrostatic potential (MEP) surfaces of **1** at a selected level of theory.

Synthesized polymers as metallodrugs may serve as potential anti-cancer agents, as suggested by other reports [[91], [92], [93], [94], [95], [96], [97], [98], [99], [100], [101]]. Therefore, we conducted an in silico study to explore the anti-prostate cancer properties of this polymer in a water medium at 310 K (body temperature). The exact energy level of the Mn(II) polymer was calculated via the optimization process, which was executed without any symmetry constraints (C1 symmetry). Confirmation of the optimized geometries being in local minima was provided by the absence of imaginary frequencies in the vibrational frequency test. The investigation of the interactions between the calculated ground state of Mn(II) polymer, serving as ligands, and the AR, was conducted using a B97/Lanl2dz level of theory to explore low-lying structures of closed shell polymer, as illustrated in Fig. 15. Additionally, molecular electrostatic potential (MEP) maps were utilized to forecast the strength of these interactions [102]. The MEP map visually represents the charge distribution related to positive and negative points indicating low and high electron density, denoted by yellow, blue, and green colors respectively for the most negative, positive, and zero electrostatic potentials, as shown in Fig. 16. Under the initially considered conditions, the positive points and low electron density of the Mn atoms revealed the electrophilic property of this metal to other parts of the compounds, aligning with expectations. This finding further underlines the high nucleophilic properties of the Mn(II) polymer and their inclination to react with the electrophilic AR.

**Fig. 17:**
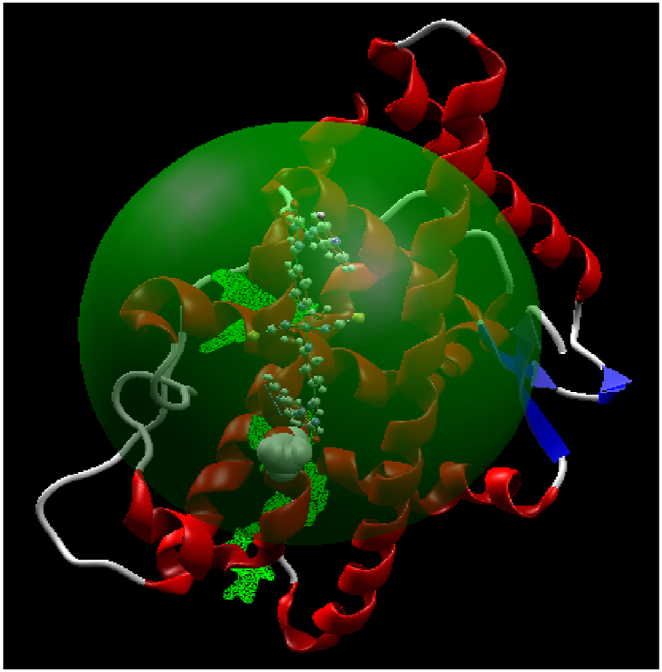
The best cavity of Mn(II) polymer and the human androgen receptor molecular simulation.

In the MM part, we optimized the geometry of the AR protein with ID: 2oz7. Subsequently, after identifying low low-lying structure, we conducted docking of Mn(II) polymer and the AR to determine optimal cavity, interaction, active sites, and type of interactions. Additionally, we re-optimized Additionally, we re-optimized the Mn(II) polymer@AR structure to achieve the local minimum of this configuration. During the geometry minimization process, the best cavity was identified based on specific Cartesian coordinates, with a radius of 15 Å, volume of 1438.2 Å³, and surface area of 2420.5 Å^2^, See Fig. 17.

**Fig. 18:**
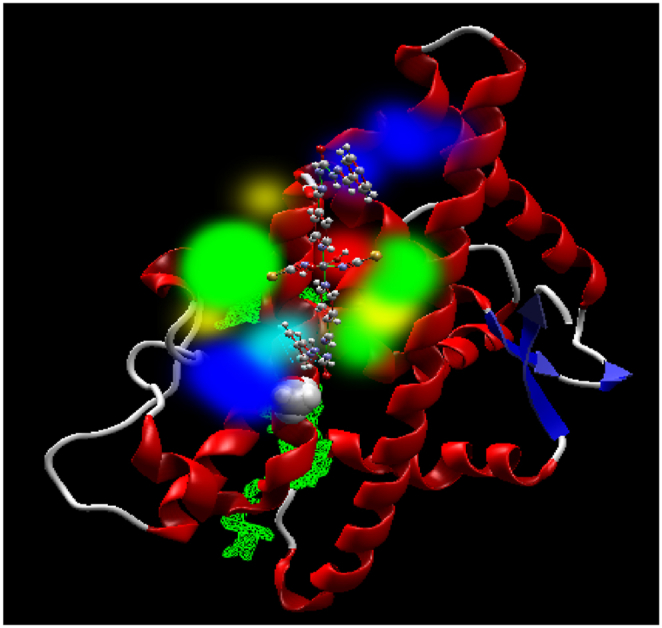
Energy map of the Mn(II) polymer at the binding cavity of the human androgen receptor.

The docking scoring results indicate a re-rank score of −89.0 kJ and a molecular interaction energy of −116.3 kJ mol^−1^, indicating a strong binding of Mn(II) polymer to the active site of the AR. This interaction is characterized by robust molecular interactions between Mn(II) polymer and with residues of the AR such as Gln (792), Val (785), His (789), and Leu (790). These interactions involve hydrogen bonding, steric interaction, and covalent bonding. The energy map of interaction in Fig. 18 illustrates these interactions, with steric interaction denoted in green, hydrogen acceptor in turquoise, hydrogen donor in yellow, and electrostatic potential in red and blue. The visualization suggests that steric interaction and electrostatic potential are the main interactions in the AR and Mn(II) polymer. These interactions in the specified cavity indicate that this polymer could be an effective anti-prostate candidate, as the AR is involved in the growth of prostate cancer cells, and these interactions suggest inhibition of prostate cancer cell growth.

## 4 Conclusions

The research focused on the synthesis and characterization of a novel compound, referred to as **1**. Various techniques such as SEM, FT-IR, PXRD, and elemental analysis were employed to study this compound. The crystal structure of **1** was determined using single-crystal X-ray diffraction (SCXRD), which revealed that Mn^2+^ ions are six-coordinated within the compound. Additionally, powders generated through the sonication ultrasound method exhibited the same crystalline phase as those obtained from the solvothermal method. The study also investigated the effects of time, concentration, and reactant temperature on the morphological properties of **1.** By optimizing these parameters, the researchers identified the conditions that yielded small-sized and less agglomerated nanostructure material. Specifically, the optimal conditions were found to be a temperature of 70 °C, a reaction time of 60 min, an ultrasonic irradiation power of 60 W, and a concentration of reactants of 0.1 M. These findings demonstrated the potential of the ultrasound technique for producing nanostructures of **1**. The crystallographic data for the reported structure were deposited with the Cambridge Crystallographic Data Center as supplementary publication No. CCDC-2149580, ensuring the availability and reproducibility of the results. Furthermore, the research investigated the Hirshfeld surface analysis of **1**, further confirming the potential of ultrasound for obtaining nanostructures of Mn_3_O₄. The study used the B97/LANL2DZ level of theory to obtain the local minimum of the Metal Organic Coordination Polymer (MOCP). The utilization of molecular docking methodologies demonstrated that the best cavity of the human androgen receptor possessed an interaction energy of −116.3 kJ mol^−1^. The findings indicated that steric interaction and electrostatic potential are the primary interactions in AR polymer and Mn(II). These interactions within the specified cavity suggest that this polymer could be a promising anti-prostate candidate. As AR is involved in the growth of prostate cancer cells, these interactions imply the potential to inhibit the growth of prostate cancer cells.

## Data availability statement

We encourage all authors of articles published in MDPI journals to disclose their research data. In this section, authors should provide details on where the data supporting reported results can be accessed, including links to publicly archived datasets analyzed or generated during the study. If no new data were created, or if data is unavailable due to privacy or ethical constraints, a statement is still necessary. For suggested Data Availability Statements, please refer to the “MDPI Research Data Policies” section at https://www.mdpi.com/ethics.”

## CRediT authorship contribution statement

**Atash V. Gurbanov:** Methodology. **Fateme Firoozbakht:** Writing – original draft. **Nafiseh Pourshirband:** Writing – original draft. **Paria Sharafi-Badr:** Software, Methodology. **Payam Hayati:** Writing – original draft, Software, Project administration, Methodology. **Bagher Souri:** Writing – original draft. **Fazlolah Eshghi:** Writing – original draft, Software. **Werner Kaminsky:** Software. **Ghodrat Mahmoudi:** Methodology, Investigation. **Francis Verpoort:** Project administration, Investigation, Formal analysis. **Zohreh Mehrabadi:** Writing – review & editing, Writing – original draft, Software, Methodology.

## Declaration of competing interest

The authors declare that they have no known competing financial interests or personal relationships that could have appeared to influence the work reported in this paper.
