# Corrigendum to “A new 1D Mn(II) coordination polymer: Synthesis, crystal structure, hirshfeld surface analysis and molecular docking studies” [Heliyon Volume 10, Issue 8, April 2024, Article e29565]

**DOI:** 10.1016/j.heliyon.2025.e43586

**Published:** 2025-07-05

**Authors:** Atash V. Gurbanov, Fateme Firoozbakht, Nafiseh Pourshirband, Paria Sharafi-Badr, Payam Hayati, Bagher Souri, Fazlolah Eshghi, Werner Kaminsky, Ghodrat Mahmoudi, Francis Verpoort, Zohreh Mehrabadi

**Affiliations:** a Centro de Química Estrutural, Institute of Molecular Sciences, Instituto Superior T'ecnico, Universidade de Lisboa, Av. Rovisco Pais, 1049-001, Lisboa, Portugal; b Excellence Center, Baku State University, Z. Khalilov Str. 23, AZ, 1148, Baku, Azerbaijan; c Department of Chemistry, University of Isfahan, Isfahan, 81746-73441, Iran; d Department of Chemistry, Shahreza Branch, Islamic Azad University, P.O. Box 311-86145, Shahreza, Isfahan, Iran; e Department of Pharmacognosy and Pharmaceutical Biotechnology, School of Pharmacy, Iran University of Medical Sciences, Tehran, Iran; f Organic and Nano Group (ONG), Department of Chemistry, Iran University of Science and Technology (IUST), PO Box 16846-13114, Tehran, Iran; g Department of Chemistry, Faculty of Sciences, University of Sistan and Baluchestan, Zahedan, Iran; h Department of Chemistry, College of Sciences, Shiraz University, Shiraz, Iran; i X-ray Crystallography Laboratory, University of Washington, United States; j Department of Chemistry, Faculty of Science, University of Maragheh, P.O. Box 55136-83111, Maragheh, Iran; k Chemistry Department, Faculty of Engineering and Natural Sciences, Istinye University, Sarıyer, Istanbul, 34396, Turkey; l State Key Laboratory of Advanced Technology for Materials Synthesis and Processing, Wuhan University of Technology, Wuhan, 430070, China; m Department of Chemistry, Firoozabad Branch, Islamic Azad University, Firoozabad, Iran; n Western Caspian University, Istiqlaliyyat Street 31, AZ 1001, Baku, Azerbaijan

In this article, the authors have included the references [7] and [100] which have been found to be redundant to the manuscript:

*[7] X. Zhu, T. Wu, Y. Chi, Y. Ge, B. Wu, M. Zhou, F. Zhu, M. Ji, L. Cui, Pyroptosis induced by enterovirus A71 infection in cultured human neuroblastoma cells, Virology 521 (2018) 69–76,* https://doi.org/10.1016/j.virol.2018.05.025 *.*

*[100] Y. Yang, Sh M. Zhang, Y.Q. Zhang, L. Chen, T. Xiong, A new mixed-ligand Mn(II) coordination polymer: protective and nursing values on renal calculus via reducing the Ca2+ concentration in urine, Prog. React. Kinet. Mech. 47 (2010) 1–10, https://doi.org/10.1177/14686783221090373.*

In agreement with the editor's recommendation, the authors have replaced reference [7] and removed reference [100]. The correct version of reference [7] should be as below:

*[7] Gohy, Jean-François. "Metallo-supramolecular block copolymer micelles." Coordination Chemistry Reviews 253, no. 17–18 (2009): 2214–2225. https://doi.org/10.1016/j.ccr.2008.11.017.*

The correct version of the last part of the reference list should be as below:

*[100] Y. Yang, Sh M. Zhang, Y.Q. Zhang, L. Chen, T. Xiong, A new mixed-ligand Mn(II) coordination polymer: protective and nursing values on renal calculus via reducing the Ca2+ concentration in urine, Prog. React. Kinet. Mech. 47 (2010) 1–10, https://doi.org/10.1177/14686783221090373.*

*[102] G. Tasi, I. Palinko, L. Nyerges, P. Fejes, H. Foerster, Calculation of electrostatic potential maps and atomic charges for large molecules, J. Chem. Inf. Comput. Sci. 33 (1993) 296–299.*

The ***authors*** apologize for the errors.

## Declaration of competing interest

The authors declare that they have no known competing financial interests or personal relationships that could have appeared to influence the work reported in this paper.

