# Supplementary material for: A new 1D Mn(II) coordination polymer: Synthesis, crystal structure, hirshfeld surface analysis and molecular docking studies
Source: Heliyon. 2024 Apr 20;10(8):e29565. doi: 10.1016/j.heliyon.2024.e29565 (PMC11063412; doi:10.1016/j.heliyon.2024.e29565)
Supplement: Multimedia component 1 [file mmc1.docx]

**Supporting Information**

### [**A new 1D Mn (II) coordination polymer: Synthesis, crystal structure, Hirshfeld surface analysis and molecular docking studies**](https://www.sciencedirect.com/science/article/pii/S0020169320310331?casa_token=gsvYu8e3aVAAAAAA:N4TKxiy2nwKCZp42L3A-XBOAcU-jOY_qJF_hYiNowfF7ShzDLaGh8RYDH5p2uMcigw1O1g9NYA)

Atash V. Gurbanov ^a,b^, Fateme Firoozbakht^c^, Nafiseh Pourshirband ^d^, Paria Sharafi-Badr ^e^, Payam Hayati ^f,^*, Bagher Souri ^g^, Fazlolah Eshghi ^h^, Werner Kaminsky ^i^, Ghodrat Mahmoudi ^j,k^ **, Francis Verpoort ^l,^***, Zohreh Mehrabadi ^m^

^a^ Centro de Química Estrutural, Institute of Molecular Sciences, Instituto Superior Técnico, Universidade de Lisboa, Av. Rovisco Pais, 1049-001 Lisboa, Portugal

^b^ Department of Chemistry, Baku State University, Z. Khalilov Str. 23, AZ 1148 Baku, Azerbaijan

^c^ Department of Chemistry, University of Isfahan, Isfahan 81746-73441, Iran

^d^ Department of Chemistry, Shahreza Branch, Islamic Azad University, P.O. Box 311-86145, Shahreza, Isfahan, Islamic Republic of Iran ^e^ Department of Pharmacognosy and Pharmaceutical Biotechnology, School of Pharmacy, Iran University of Medical Sciences, Tehran, Iran

^f^ Organic and Nano Group (ONG), Department of Chemistry, Iran University of Science and Technology (IUST), PO Box 16846-13114, Tehran, Iran

^g^ Department of Chemistry, Faculty of Sciences, University of Sistan and Baluchestan, Zahedan, Iran. ^h^ Department of Chemistry, College of Sciences, Shiraz University, Shiraz, Iran

^i^ X-ray Crystallography Laboratory, University of Washington, United States

^j^ Department of Chemistry, Faculty of Science, University of Maragheh, P.O. Box 55136-83111, Maragheh, Iran.

^k^ Chemistry Department, Faculty of Engineering and Natural Sciences, Istinye University, Sarıyer, Istanbul 34396, Turkey

^l^ State Key Laboratory of Advanced Technology for Materials Synthesis and Processing, Wuhan University of Technology, Wuhan 430070, PR China

^m^ Department of Chemistry, Firoozabad Branch, Islamic Azad University, Firoozabad, Iran

*E-mail: [payamhayati@yahoo.com](mailto:payamhayati@yahoo.com). **E-mail:

[gmahmoodi@gmail.com](mailto:gmahmoodi@gmail.com***E-mail)

[***E-mail](mailto:gmahmoodi@gmail.com***E-mail): [francis@whut.edu.cn](mailto:francis@whut.edu.cn)


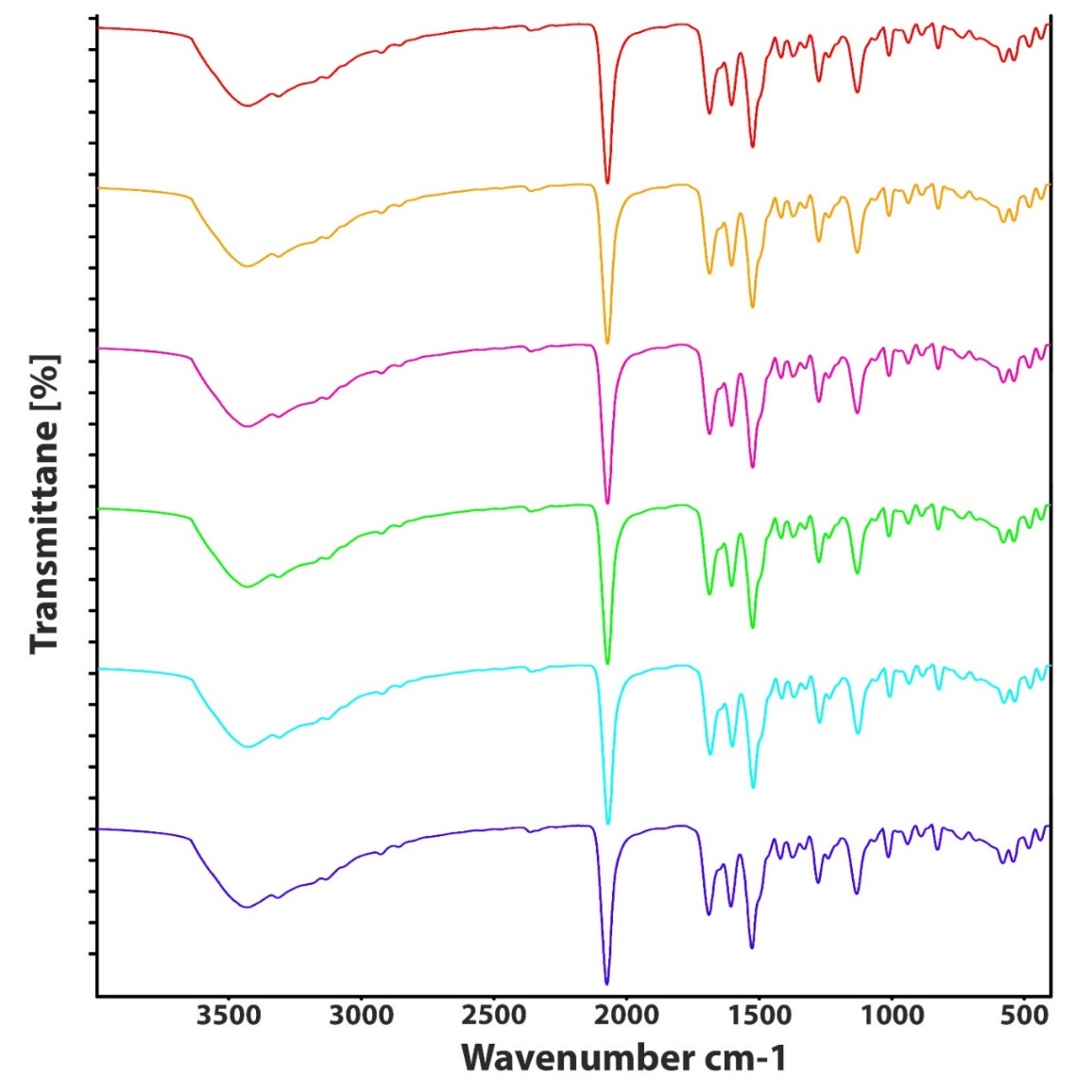
**Fig S1.** The IR spectra of, 1-1 obtained by ultra-sonication (red line), 1- 2 (brown line), 1-3 (pink line), 1-4 (green line), 1-5 (light blue line) as well as (simulated from SCXRD data of **1).**

**Fig S2.** a) Simulated pattern based on single crystal data of a) 1, b) 1-1, c) 1-2, d) 1-3, e) 1-4 and f) 1-5.

**Table S1**. Crystallographic data for 1.

| [C_15_H_12_MnN_8_OS_2_·CH_4_O·2(HO)](about:blank) | Empirical formula |
| --- | --- |
| 505.44 g/mol | Formula weigh |
| 292 K | Temperature |
| 0.71073 Å | Wavelength |
| Triclinic | Crystal system |
| *P_-1_* | Space group |
| a = 8.7970(6) Å, α = 103.154(3)°  b = 11.0890(10)Å, β = 104.936(2)°  c = 14.5830(14)Å, γ= 103.461(4)° | Unit cell dimensions |
| 1272.61(19) Å^3^ | Volume |
| 2 | Z |
| [0.](about:blank)4 × [0.](about:blank)3 × [0.](about:blank)3 (mm) | Crystal size |
| [0.935](about:blank) Mg/m^3^ | Absorption coefficient |
| 518 | F(0 0 0) |
| 3 to 20° | Theta range for data collection |
| 0.72 mm^-1^  -10 ≤ h ≤ 9  -13 ≤ k ≤ 13  -17 ≤ l ≤ 17 | μ  Index ranges |
| 0.602 Å^−1^ | (sin θ/λ)max |
| 0.814 ° | Theta(max) |
| [Mo Kα](about:blank)  Full–matrix least–squares on F^2^  1.007 | Radiation type  Refinement method  Goodness- of- fit on F^2^ |
| R[F^2^> 2σ(F^2^)]=[0.058](about:blank)  wR(F^2^)=[0.179](about:blank)  S=[0.99](about:blank) | Refinement |
| 0.052  [0.40](about:blank), [−0.33](about:blank)  2149580 | Rint  Δρ_max_, Δρ_min_ (e Å^−3^)  CCDC no. |
